# Bronchopulmonary dysplasia: clinical aspects and preventive and therapeutic strategies

**DOI:** 10.1186/s12967-018-1417-7

**Published:** 2018-02-20

**Authors:** Nicola Principi, Giada Maria Di Pietro, Susanna Esposito

**Affiliations:** 1 Università degli Studi di Milano, Milan, Italy; 2 Pediatric Clinic, Department of Surgical and Biomedical Sciences, Università degli Studi di Perugia, Piazza Menghini 1, 06129 Perugia, Italy

**Keywords:** Bronchopulmonary dysplasia, Lung, Prematurity, Preterm neonates, Respiratory disease, Respiratory tract infection

## Abstract

**Background:**

Bronchopulmonary dysplasia (BPD) is the result of a complex process in which several prenatal and/or postnatal factors interfere with lower respiratory tract development, leading to a severe, lifelong disease. In this review, what is presently known regarding BPD pathogenesis, its impact on long-term pulmonary morbidity and mortality and the available preventive and therapeutic strategies are discussed.

**Main body:**

Bronchopulmonary dysplasia is associated with persistent lung impairment later in life, significantly impacting health services because subjects with BPD have, in most cases, frequent respiratory diseases and reductions in quality of life and life expectancy. Prematurity per se is associated with an increased risk of long-term lung problems. However, in children with BPD, impairment of pulmonary structures and function is even greater, although the characterization of long-term outcomes of BPD is difficult because the adults presently available to study have received outdated treatment. Prenatal and postnatal preventive measures are extremely important to reduce the risk of BPD.

**Conclusion:**

Bronchopulmonary dysplasia is a respiratory condition that presently occurs in preterm neonates and can lead to chronic respiratory problems. Although knowledge about BPD pathogenesis has significantly increased in recent years, not all of the mechanisms that lead to lung damage are completely understood, which explains why therapeutic approaches that are theoretically effective have been only partly satisfactory or useless and, in some cases, potentially negative. However, prevention of prematurity, systematic use of nonaggressive ventilator measures, avoiding supraphysiologic oxygen exposure and administration of surfactant, caffeine and vitamin A can significantly reduce the risk of BPD development. Cell therapy is the most fascinating new measure to address the lung damage due to BPD. It is desirable that ongoing studies yield positive results to definitively solve a major clinical, social and economic problem.

## Background

Preterm birth has been associated with an increased risk of early and late severe clinical problems, including poor adult health. Complications are very common in extremely preterm (EPT; i.e., < 28 weeks of gestational age) and very preterm infants (VPT; i.e., 28–31 weeks of gestational age), but complications can also occur in modestly preterm infants (MPT; i.e., ≥ 32 weeks of gestational age) [1–3]. Together with chronic neurological diseases, chronic respiratory problems due to bronchopulmonary dysplasia (BPD) are the most common long-term complications of prematurity [4, 5]. BPD is the result of a complex process in which several prenatal and/or postnatal factors interfere with lower respiratory tract development, leading to a severe, lifelong disease.

When BPD was identified as a clinical problem, most patients with EPT and VPT did not survive. This clinical condition was diagnosed mainly in MPT and was considered the consequence of an inadequate therapeutic approach to neonatal respiratory distress syndrome (RDS) [6]. RDS is a respiratory disorder chiefly of newborn premature infants that is characterized by deficiency of the surfactant coating the inner surface of the lungs resulting in labored breathing, lung collapse, and hypoxemia. When survival of EPT and VPT became more common because of the introduction of prenatal steroid administration, postnatal surfactant supplementation, and safer ventilator measures, it was shown that BPD had a frequency inversely correlated with gestational age and could be associated with inadequate postnatal therapy only in a minority of cases. By contrast, it was shown that, in most cases, BPD was the consequence of a deviation from the normal lung developmental pattern, in which prematurity per se and a number of potentially interfering factors, including genetics, could play causative roles [7]. However, independent of its origin, it is presently clearly established that BPD is associated with persistent lung impairment later in life, significantly impacting health services because subjects with BPD have, in most cases, frequent respiratory diseases and reductions in quality of life [8].

Fortunately, in recent years, the characteristics of lung development have been better defined. Moreover, most of the factors that favour BPD development have been identified [6–10]. Despite the fact that not all of the problems related to the relationships of foetal and neonatal lung damage with later respiratory problems have been clarified, a number of effective preventive and therapeutic management strategies with potential reductions in BPD complications have been developed. In this review, what is presently known regarding the impact of BPD on long-term pulmonary morbidity and mortality and the available preventive and therapeutic strategies are discussed.

## The two types of bronchopulmonary dysplasia (BPD)

According to Jobe [11], “the injury resulting in BPD likely begins as altered lung development before delivery in many infants and can be initiated by resuscitation at birth and then amplified by postnatal exposures”. The timing, type and duration of exposures, together with the genetic characteristics of the child, influence the pattern of lung damage that can occur. BPD as initially described (i.e., old BPD) was considered the result of an aggressive mechanical ventilator approach in terms of peak pressures and oxygen concentrations on a relatively mature lung lacking surfactant (i.e., ≥ 32 weeks of gestation) [12]. Several experimental studies have clearly shown that ventilation with high positive pressure and excess volume can lead to alveolar damage and severe local inflammation, with higher than normal tumour necrosis factor-α, interleukin (IL)-1β, interleukin-6, and macrophage inflammatory protein-2 concentrations in the lung [13, 14]. Moreover, aggressive ventilation has been associated with reactive oxygen species (ROS) production. Maturation of antioxidant enzyme capacities is gradual during foetal life, and some enzymes, such as catalase and copper–zinc superoxide dismutase, are significantly induced by breathing air after birth [15], indicating that even a slight increase in reactive oxygen species in moderately preterm babies can lead to lung damage [12–14]. Moreover, in experimental animals, supraphysiological oxygen concentrations during the first days of life were found to be able to compromise alveolarization and lead to right ventricular hypertrophy, vascular remodelling, and the development of small airways disease, smooth-muscle hypertrophy, and oxidative stress [16]. Similar findings were shown in lung tissue sections of neonates born before term who developed respiratory distress syndrome and were treated with high oxygen concentrations and aggressive ventilation [17].

Thanks to improvements in neonatal intensive care, both the pathogenesis and the histology of BPD have changed, and a new type of BPD has developed. More preterm infants born in the early stages of lung development presently suffer from BPD, with a frequency inversely correlated with gestational age. Most cases of BPD occur in babies born before the 32nd week of gestation, when the lung is in the canalicular (from the 17th to 26th week of gestation) or saccular (from the 27th to 36th week of gestation) stage of development [17]. Particularly in EPT, the lung structure is very immature. The respiratory bronchioles, precapillaries and mucous glands in the bronchi are not completely developed, the interstitium has not thinned adequately to form the blood–air barrier, and surfactant production by the lung epithelial cells has not started. Moreover, when preventive and therapeutic measures, such as antenatal steroid and postnatal surfactant administration, are used, even subjects born several weeks before term often develop mild to moderate respiratory problems. Unfortunately, several factors can interrupt lung development, reducing pulmonary microvascular growth and alveolarization. Practically, when foetal or postnatal factors, together with genetic predisposition, interact with a lung in early development, the development is altered, and a new type of BPD (i.e., new BPD) occurs. The already cited mechanical trauma and oxygen toxicity again play roles, but several different exposures, both prenatal and/or postnatal, are involved in this altered lung development. Maternal smoking and hypertension have been found to be associated with a twofold increase in the odds of developing new BPD [18]. Infections can have similar effects, although results of the studies specifically planned in this regard were in some cases partly conflicting. The relationship seems well defined for postnatal infections. Lapcharoensap et al., in a retrospective, population-based cohort study, examined whether reductions in rates of nosocomial infection were associated with changes in rates of BPD [19]. Adjusted rates of nosocomial infections and BPD from a baseline period (2006–2010) were compared with a later period (2011–2013). A total of 22,967 infants were included in the study. From the first to second period, the incidence of nosocomial infections decreased from 24.7 to 15%, and BPD decreased from 35 to 30%. Adjusted hospital rates of BPD and nosocomial infections were correlated positively with a calculated 8% reduction in BPD rates attributable to reductions in nosocomial infections. Similar findings were reported when the role of sepsis was considered [20]. More controversy, however, exists regarding the relevance of chorioamnionitis. In a meta-analysis of 57 studies enrolling more than 15,000 subjects, it was found that, considering together all the cases of chorioamnionitis regardless of type, infants born to mothers with this condition had a risk of developing BPD only slightly higher than that of controls (weighted mean differences [WMD] 1.89; 95% confidence interval [CI] 1.5–2.3). The association remained significant for histological (WMD 2.19; 95% CI 1.7–2.7) but not for other types of chorioamnionitis. Moreover, the conclusion of the meta-analysis was considered highly debatable mainly because most of the included studies had poor methodological quality [21]. However, a recent retrospective cohort study, in which it was evaluated whether chorioamnionitis affected the incidence of BPD after accounting for the increased risk of death, showed that prenatal infection was significantly associated with BPD and perinatal death (odds ratio [OR] 5.18; 95% CI 4.39–6.11] [22]. A role also seemed to be played by growth restriction. Neonates small for their gestational age were found to have a more than a two times higher risk (OR 2.73; 95% CI 2.11–3.55) of BPD development than subjects with weight appropriate for their gestational age [23]. Moreover, poor nutrition in the first days of life has been found to be associated with an increased risk of BPD [24]. Finally, genetics might contribute to BPD development. In a study conducted in twins [25], it was shown that, among monozygotic twin pairs, the observed concordance for BPD was significantly higher than the expected concordance. After controlling for covariates, genetic factors accounted for 53% of the variance in predisposition to BPD. Moreover, it was reported that the *SPOCK2* gene might be considered a new possible candidate susceptibility gene for BPD because its lung expression pattern points towards a potential role in alveolarization [26]. However, the role of genetics requires further evaluation. A large genome-wide association study enrolling more than 1700 neonates did not identify genomic loci or pathways that could account for the previously described heritability of BPD [27].

In comparison with old BPD, new BPD has more severe alveolar damage, fewer severe arterial/arteriolar vascular lesions, and negligible airway epithelial lesions [28–31].

## Definition and prevalence of bronchopulmonary dysplasia (BPD)

Initially, BPD was defined simply by considering the need for oxygen 28 days after birth [6]. However, in a study in which the follow-up records of 605 infants with birth weights less than 1500 g, with data available for 2 years after birth, were examined, it was shown that the need for oxygen at 28 days was a good predictor of abnormal findings in infants with a gestational age ≥ 30 weeks at birth, but it became less useful as gestational age decreased. Irrespective of gestational age at birth, the requirement for additional oxygen at 36 weeks of corrected postnatal gestational age was found to be a better predictor of respiratory problems. Moreover, normal respiratory outcomes were demonstrated in 90% of infants not receiving oxygen at this corrected gestational age [32].

However, a definition based on the need for oxygen or the tolerance of room air at 36 weeks of life was also not considered fully appropriate because oxygen administration might vary according to clinical practice among different centres, and BPD severity was not categorized. Several attempts to propose more appropriate definitions have been undertaken, and some of them were used clinically and for research purposes [7]. The most frequently used definition differentiates EPT from VPT. All cases are examined at 28 days, whereas a second evaluation is performed at 36 weeks for EPT and at 56 days for VPT. BPD is diagnosed in all preterm infants who needed oxygen at 28 days. However, BPD is considered mild if, at the time of the final evaluation, the child can tolerate room air, moderate if he or she requires < 30% oxygen and severe if > 30% oxygen is required. Need for nasal CPAP or mechanical ventilation further supports the definition of severe BPD [33].

However, even this classification cannot be considered fully satisfactory because it considers only the simplest therapeutic aspects and not the multiplicity of measures presently used in neonatal intensive care units. Moreover, it seems likely that the risk of developing long-term lung dysfunction could be more precisely predicted were circulatory problems that follow premature birth considered. As evidenced by Day and Ryan [34], subclinical vascular disease might play a role in influencing lung development, and both prenatal and postnatal factors might differently influence angiogenesis. Increased knowledge of these aspects of lung development might lead to the identification of a number of biomarkers able to indicate the degree of damage and the risk of later respiratory problems. Supporting this hypothesis is the evidence that prematurity is associated with suppression of vascular endothelial growth factor and induction of angiopoietin 2 [35–37]. Moreover, prematurity and mechanical ventilation have been strictly correlated with the regulation of angiogenesis through the activity of endoglin and matrix metalloproteinase 9 [38, 39].

Regarding frequency, the different definitions used to identify BPD make it difficult to evaluate the real incidence of the condition and to compare variations over time. However, several studies have suggested that, with the increase in survival rates of EPT and VPT, the incidence of children with BPD has remained quite similar to that initially calculated, although mortality has decreased due to the improved prophylactic and therapeutic measures presently available. Recent evaluations in the USA have indicated that BPD develops in approximately 10% of VPT and in 40% of EPT when the need for oxygen at 36 weeks of postmenstrual age (i.e., the time elapsed between the first day of the last menstrual period and birth plus the time elapsed after birth) is considered to identify BPD [38], indicating that, in the USA each year, approximately 5000–10,000 new cases of the disease are diagnosed. In Europe, it was found that 10–20% of all infants born between 23 and 31 weeks of postmenstrual age developed BPD [40, 41].

## Long-term outcomes of bronchopulmonary dysplasia (BPD)

Prematurity per se is associated with an increased risk of long-term lung problems. However, in children with BPD, impairment of pulmonary structures and function is even greater, although the characterization of long-term outcomes of BPD is difficult because the adults presently available to study have received outdated treatment.

### Persistence of lung structural alterations

Bronchopulmonary dysplasia is associated with an increased risk of persistent lung anatomical and functional alterations in patients with both old and new BPD. Regarding anatomical damage, several studies have shown that most subjects with old BPD, when evaluated during adolescence or adulthood, had significant alterations on chest computed tomography (CT) scans [31, 42–45]. Good examples in this regard are the studies by Aukland et al. [42] and Wong et al. [43]. The first authors used high-resolution CT (HRCT) to study the lung characteristics of a cohort of 10- to 18-year-old subjects born at a gestational age of ≤ 28 weeks or with a birth weight ≤ 1000 g who received prolonged oxygen treatment or were diagnosed with old BPD. The presence of the following variables was studied: linear and subpleural triangular opacities, decreased lung attenuation (on inspiration and/or expiration), bronchial wall thickening, bronchus-to-artery-diameter ratio, bullae, emphysema, collapse/consolidation and bronchiectasis. Each lobe was scored according to either the presence or absence of each of the aforementioned variables in any lung segment. Pathological findings were detected in 86% of children and adolescents, mainly the presence of linear/triangular opacities, which were seen in 82% of cases. Compared with subjects without BPD or with only mild BPD, subjects with a history of moderate or severe BPD had significantly higher total HRCT scores (mean 3.0 vs 5.2; p  =  0.009), as well as more opacities (p  =  0.035) and hypoattenuated areas (p  =  0.007). The very high frequency of HRCT pathological findings was also evidenced by Wong et al. [43], who studied adults born preterm and for whom a diagnosis of BPD had been made. The only difference with the study by Aukland et al. [42] was the frequency with which the various HRCT findings were detected. In the study by Wong et al. [43], among HRCT alterations, pulmonary emphysema was the most common and was detected in 71% of cases.

Studies regarding persistent anatomical alterations in subjects with new BPD have been few. Moreover, compared with studies conducted in subjects with old BPD, they included younger subjects. However, the conclusions were quite similar, clearly showing that, despite advances in neonatal care, persistent lung lesions remained very common. Mahut et al. reported the HRCT results of 41 very low birth weight infants with new BPD between 10 and 20 months old [46]. Despite advances in neonatal care, many CT findings in infants with BPD were similar to those observed in the pre-surfactant era and were still associated with the durations of supplemental oxygen and mechanical ventilation. All of the CT scans were abnormal, with linear and triangular subpleural opacities and hyperlucent areas seen on the vast majority of scans.

### Persistence of lower respiratory tract functional abnormalities

Together with structural persistent modifications, BPD can be associated with significant functional alterations of the lower respiratory tract. This association was shown in younger infants, in school-aged children, in adolescents and in adults, clearly showing that intrauterine and neonatal lung damage can persist and worsen into adulthood. Respiratory impairment was found as a consequence of both old and new BPD. Comparisons between studies are difficult because of the different criteria used to define BPD and, among studies of only old or new BPD, because of variations in the study design, age of enrolled patients and study end points used to measure pulmonary outcomes. However, in most of the cases, a significant impact of BPD was shown. Regarding old BPD, Hakulinen et al. examined 42 children 6–9 years old, among whom 10 had a history of BPD, 19 had received neonatal respiratory treatment but had no history of BPD, and 13 had no severe neonatal respiratory problems [47].

The BPD children had markedly lower specific airway conductance and larger residual volumes than the full-term control group, although there were no significant differences in spirometric measurements. By contrast, pulmonary function variables in prematurely born children who did not receive ventilator treatment did not differ from those in the full-term control group. Northway et al. compared in a case–control study the pulmonary function of 52 adolescents and young adults born prematurely (26 with BPD) with that of 53 healthy controls born at term [48]. The authors reported that 68% of the subjects with BPD had airway problems [lower forced vital capacity (FVC), lower forced expiratory volume in 1 s (FEV1) and lower forced expiratory flow between 25 and 75% of vital capacity (FEF25%–75%) than individuals included in the preterm group without BPD and term controls. Furthermore, 24% of the subjects with old BPD had fixed airway obstruction, and 52% had reactive airway disease, as indicated by their responses to the administration of methacholine or a bronchodilator.

Details on the short- and long-term respiratory outcomes of new BPD can be derived from the paper by Islam et al., who reviewed all of the literature on this topic published during or since 1990 [49]. Because the greatest part of the studies analysed by these authors were conducted after the routine use of prenatal steroids and the availability of surfactant, it is highly likely that the conclusions of this review apply mainly to new BPD. Airway obstruction was the most important finding in most of the studies, and it was demonstrated in the first controls after the few months of life and persisted over time. Baraldi et al. reported that children with BPD, examined at 2 years of age, showed a substantial improvement in pulmonary mechanics but still presented with a substantially low FEF [50]. Robin et al. compared the respiratory function of 28 preterm infants with BPD and 41 healthy, full-term subjects and found that BPD was associated with mild to moderate airflow obstruction and air trapping [51]. In particular, these authors reported decreased FEFs, including FEV in 0.5 s, forced FEF at 75% and FEF25–75% in children with disease, compared to controls. Fakhoury et al. prospectively and longitudinally measured lung function in a cohort of 44 children with BPD (mean gestational age 25.6 weeks and mean birth weight 0.767 kg) during their first 3 years of life [52]. Although lung volumes increased with time, persistent flow limitation was evidenced. At 6, 12 and 24 months, low partial expiratory airflow, measured by maximum flow at functional residual capacity (VmaxFRC), was shown, without any significant increase over time.

Compromised pulmonary function has also been demonstrated in older children, although evaluation of the role of BPD in conditioning alterations is difficult to ascertain. A meta-analysis of 59 studies reported that percent of FEV1% was reduced in preterm-born survivors, even when they did not develop BPD [53]. However, Hacking et al. compared respiratory function at 8 years in 201 extremely low birth weight (ELBW)/EPT and 199 randomly selected normal birth weight/full term controls [54]. FEV1 and FEF25–75% were found to be significantly lower in patients than in controls. However, within the ELBW/EPT cohort, children who had BPD in the newborn period had significant reductions in both the FEV1 (− 0.76 standard deviation [SD]) and FEF25–75% (− 0.58 SD), compared with those who did not have BPD.

### Clinical problems

#### Hospital re-admission

BPD is a risk factor for re-hospitalization. Several studies have reported that preterm infants are re-hospitalized more frequently than term infants. However, the prevalence of re-hospitalization is even greater in those with BPD. Lamarche-Vadel et al. estimated the re-hospitalization rate of 376 patients with EPT during their first 9 months after discharge and found that 47.3% were re-admitted at least once in 55% of the cases for respiratory disorders [55]. However, the re-hospitalization rate was higher for children who had had BPD (OR 2.2; 95% CI 1.3–3.7).

Ralser et al. reported the rate of readmission of 377 children born before 32 weeks of gestational age and found that 151 of them (40.1%) in the first and 93 of them (24.7%) in the second year of life were hospitalized [56]. Respiratory disorders accounted for 42.1 and 47.4% of total readmissions, respectively, in the first and second years of life. BPD was a risk condition relevant to readmission in both years of the study period. Greenough et al. conducted a study in 235 infants with a median gestational age of 27 weeks [57]. They demonstrated that, despite routine use of antenatal steroids and postnatal surfactant, patients with BPD, particularly those who received home oxygen treatment, showed high rates of re-hospitalization after discharge from the neonatal care unit (median of 2 new admissions per patient).

#### Risk of respiratory tract infections and asthma-like symptoms

In preterm infants with BPD, the risk of respiratory infections is greater than in preterm infants without BPD, who in turn suffer from respiratory infections more frequently than term infants [58]. Infections can lead to even greater lung damage, as evidenced by several epidemiological evaluations and animal studies. Moreover, common respiratory infections can cause very severe respiratory disease leading to hospitalization in most cases. A good example in this regard was provided in the study by Groothuis et al. [59]. These authors studied morbidity due to respiratory syncytial virus (RSV) infection during an epidemic in children with BPD. RSV disease was diagnosed in 59% of old BPD patients, with 69% of them requiring hospitalization that lasted more than 7 days in approximately two-thirds of cases. By contrast, hospitalization rates for normal children ranged from 1 in 50 to 1 in 500, and the usual duration of hospitalization was four to 7 days. However, modern approaches to the prevention of BPD development and the use of prophylaxis against RSV have significantly reduced the clinical problems due to this virus in children with new BPD. A recent evaluation of RSV hospitalization rates in the USA between 1998 and 2008 revealed that, in the 11-year period, the predicted rate of RSV hospitalization statistically significantly decreased by 48% (from 93.78 to 49.06 per 1 million children; p = 0.013).

Regarding asthma-like signs and symptoms, it has been reported that children with EPT at 11 years of age suffered from clinically evident asthma in 25% of cases and had abnormal spirometry in more than 50%. However, despite similar clinical features, allergic asthma and asthma following BPD are different. Patients with allergic asthma have an increased prevalence of atopy, eosinophilic inflammation and high levels of exhaled nitric oxide. Children with BPD have neutrophilic airway inflammation and lower values of exhaled nitric oxide and exhaled breath temperature [60]. The airway obstruction is only partially reversed by beta2-agonists, the response to inhaled corticosteroids is poorer, and the acute exacerbations are fewer because patients with BPD suffer from fixed airway narrowing, as evidenced by HRTC studies [61–63].

Children with BPD might be intolerant to exercise due to abnormal bronchial function. Karila et al. reported that children with BPD examined at 7–14 years old had ventilatory limitations on exercise, with greater use of the ventilatory reserves (p < 0.01) and lower maximal ventilation (p < 0.01) and tidal volume (p = 0.01) [64]. Moreover, changes in ventilation (p < 0.0001) and tidal volume (p = 0.003) during exercise were significantly smaller in the BPD group than in controls.

#### Lung arterial hypertension

The lung vascular abnormalities that accompany BPD can lead to pulmonary arterial hypertension. In a recent meta-analysis regarding studies published from January 2000 to December 2015 it was reported that this complication occurs in 17% (95% CI 12–21) of BPD cases, regardless of severity of disease. However, it seems significantly more common in moderate-severe BPD (24%; 95% CI 17–30) [65]. Early diagnosis is essential for optimal management and for improving final outcome of these patients. Although cardiac catheterization remains the gold standard for the diagnosis of pulmonary hypertension in infants, transthoracic echocardiography is the most commonly used screening tool for this purpose because it is a noninvasive techniques and permits a readily available assessment of heart function. A retrospective review [66] of data from 25 children < 2 years of age with a diagnosis of BPD revealed that, compared with cardiac catheterization, measurements of systolic pulmonary artery pressure with echocardiography diagnosed correctly the presence or absence of pulmonary hypertension in 79% of the cases. However, severity was assessed correctly in only 47% of the children. Cardiac CT and magnetic resonance can offer details of right ventricle and pulmonary vasculature characteristics, further improving correct diagnosis of pulmonary hypertension [67]. Management is mainly directed to reduce hypertension with vasodilators. Oral sildenafil is the drug of choice. Prostacyclin analogues such as epoprostenol, given by inhalation or intravenously, and endothelin antagonists, such as oral bosentan, have been studied in a limited number of infants showing promising results. Finally, intravenous milrinone, a phosphodiesterase III inhibitor, is a possible alternative, especially in children that have poor response to previously reported drugs. However, as hypoxemia and hypercarbia and poor nutrition status are strictly related to progression of pulmonary hypertension, hypoxemic episodes have to be avoided or immediately treated and adequate nutritional support has to be assured [67]. Finally, use of nitric oxide is debated. However, administration of this gas has been suggested in preterm infants with premature or prolonged rupture of membranes, oligohydramnios and pulmonary hypoplasia as in these patients undetectable nitrite/nitrate levels in airway samples have been reported and this might indicate a reduced nitric oxide production playing a role in the pathogenesis of hypoxic respiratory failure and BPD [68, 69].

## Prevention and treatment of bronchopulmonary dysplasia (BPD)

### Prenatal prevention

Because the most severe cases of BPD occur in EPT and VPT, prevention of premature birth remains the most effective measure to reduce the risk of BPD. Use of maternal progesterone supplementation, surgical closure of the cervix with cerclage, prevention of the exposure of pregnant women to cigarette smoke, avoidance of fertility treatments when several follicles are potentially available for ovulation in order to reduce the risk of multiple pregnancies and dedicated preterm birth prevention clinics are well known measures to prevent premature births [70]. Moreover, in women at risk for premature delivery, administration of glucocorticosteroids (GCS; betamethasone, dexamethasone, or hydrocortisone) is essential to improve foetal lung maturation. In a recent Cochrane review including 30 studies of 7774 women and 8158 infants, it was reported that antenatal corticosteroids compared to placebo or no treatment reduced several problems strictly correlated with prematurity, including RDS, particularly more severe cases (average relative risk of moderate/severe RDS 0.59, 95% CI 0.38–0.91 [71]. Less important, although present, was the advantage found in the prevention of chronic lung diseases (average relative risk [RR] 0.86; 95% CI 0.42–1.79).

### Postnatal prevention and treatment

#### Ventilation

In ventilated preterm infants, permissive hypercapnia with a partial pressure of CO_2_ in the arterial blood (PaCO_2_) between 45 and 55 mmHg and a pH > 7.20 has been suggested to avoid high tidal volumes and lung overinflation. However, the effects on BPD development are modest if any [72]. By contrast, an attempt to use higher PaCO_2_ values had no positive effect [73].

It is recommended to reduce intubation as much as possible. To avoid the risk of aggressive mechanical ventilation, non-invasive respiratory ventilation strategies have been adopted, including the use of non-invasive positive pressure ventilation (NIPPV), nasal continuous positive airway pressure (NCPAP) and high flow nasal cannulas (HFNCs). A meta-analysis of 7 studies comparing ventilation strategies with or without an endotracheal tube in infants < 30 weeks of gestational age showed that avoiding the use of a tube had a small but significant beneficial impact in preventing BPD (OR 0.83; 95% CI 0.71–096) [74], indicating that these strategies should be systematically used in EPT and VPT. However, the best non-invasive method to decrease the development of BPD has not defined. Kugelman et al. reported that infants treated with NIPPV compared with those with NCPAP had reduced requirements for endotracheal ventilation and a lower incidence of BPD (2% vs 17%, p < 0.05, in the total cohort and 5% vs 33% in those < 1500 g, p < 0.05) [75]. However, a meta-analysis of 10 trials enrolling a total of 1061 infants in whom early use of NIPPV and NCPAP was compared showed that, although infants with NIPPV had a reduced risk of respiratory failure and less need for intubation, no significant differences between methods in the reduction of BPD were demonstrated [76]. Similar conclusions were reported in another meta-analysis in which the two ventilation strategies were compared after extubation [77].

#### Oxygen supplementation

The potential correlation between supraphysiological oxygen concentrations and BPD development has led to the recommendation of accurate control of oxygen administration in preterm infants. Pulse oximetry has been used to evaluate the oxygen saturation (SpO_2_) associated with the lowest risk of clinical problems in preterm infants. However, although the problem has been studied in several clinical trials, the optimal SpO_2_ for EPT and VPT has not been precisely defined. A recent evaluation of five randomized, controlled trials including more than 4800 infants, in whom lower (85–89%) versus higher (91–95%) SpO_2_ targets were compared, concluded that the lower target range did not reduce BPD, severe visual problems or rate of disability at 12–24 months. By contrast, it was associated with increased risks of death and necrotizing enterocolitis [78]. However, the quality of the available data is debated, and further studies are required to draw definitive conclusions. Some authors have indicated that, in the first few minutes of life, SpO_2_ of 70–80% might be acceptable. However, after 5 min, SpO_2_ must be maintained at between 88 and 92% with a higher alarm limit of 96%. Slightly higher SpO_2_ can be tolerated for MPT patients for whom the alarm limit of 97–98% can be accepted [79].

#### Fluid intake and nutrition

High fluid intake during the first week of life and lack of appropriate postnatal weight loss were associated in preterm infants with an increased risk of BPD development because of pulmonary congestion and oedema [80]. The same finding seems true for excess sodium intake, although in this case the difference between high and low sodium intake was not statistically significant [81]. To address the problem, EPT and VPT patients are often fluid restricted and receive no more than 120 mL/kg/day. In some cases, diuretics are added, although there is no convincing evidence of the importance of these drugs in the prevention and treatment of BPD [82]. Fluid restriction can cause problems for calorie intake that must be rapidly resolved because poor nutrition favours BPD development. It must be borne in mind that preterm infants that develop BPD have increased caloric requirements because of the increased work of breathing and because of the need to aid lung healing and growth. Feedings should achieve an intake of 150 calories/kg/day, including protein at 3.5–4 g/kg/day [83].

#### Surfactant

As previously reported, surfactant administration was one of the most important measurements capable of reducing preterm infant mortality and modifying characteristics of BPD. Early surfactant administration permitted immediate extubation to less aggressive ventilator measures, thus reducing the risk of BPD development [84]. Initially, surfactant administration was strictly associated with intubation and mechanical ventilation. When it was demonstrated that NIPPV and NCPAP were effective measures to treat RDS, non-conventional methods for surfactant administration were studied. Intratracheal catheters in infants in non-invasive ventilation were largely used and several studies have clearly evidenced that the efficacy of this procedure was even higher than that of the conventional administration of surfactant. A recent systematic review and meta-analysis [85] of the studies that have compared less and more invasive surfactant therapies has shown that the non-invasive surfactant instillation through a thin tracheal catheter in spontaneously breathing infants is significantly more effective in decreasing the risks of BPD, of death or BPD, and of NIPPV and NCPAP failure than the strategies where surfactant is administered through an endotracheal tube. Presently, surfactants purified from animal lungs, in some cases modified by the addition of specific lipids are used. These preparations are expensive to produce and supplies are limited and this explains why several attempts to produce synthetic surfactants have been made. However, due to the complex molecular structure of natural surfactants, research was not immediately effective. However, animal studies have shown that synthetic surfactants containing surfactant protein B and C analogs and a phospholipid mixture can stabilize the alveoli, measured as lung gas volumes at end expiration, even if no positive end-expiratory pressure is applied [86]. The effect on lung gas volumes seems to depend on the structure of the peptides as well as the phospholipid composition. It seems that synthetic surfactants containing two peptides and a more complex phospholipid composition could be able to replace natural surfactants within the near future, but more experiments need to be performed before any conclusion can be drawn about the ideal composition of this new generation of synthetic surfactants [86].

#### Glucocorticosteroids (GCS)

Some years ago, it was shown that GCS administration within the first 2 weeks of life to ventilated preterm infants could reduce the risk of BPD development and favour early extubation [87, 88]. Later, administration, at > 3 weeks after birth, was no more effective in BPD, although it could still facilitate earlier extubation [87, 88]. However, despite these benefits, routine use of postnatal GCS in the first days of life in children with EPT and VPT remains debated because it was found to be associated with a number of severe adverse events, including hyperglycaemia, hypertension, hypertrophic cardiomyopathy, severe retinopathy of prematurity and, mainly, cerebral palsy [87, 88].

Recently, it was suggested that, because BPD is per se associated with an increased risk of neurodevelopmental problems, a course of postnatal GCS might be of benefit in children with the highest risk of BPD [89]. Low dose hydrocortisone (1 mg/kg of hydrocortisone hemisuccinate per day divided into two doses for 7 days, followed by one dose of 0.5 mg/kg/day for 3 days) administered to children < 28 weeks of gestation, seems effective in reducing BPD without any adverse impact on neurodevelopmental outcomes [89]. Baud et al. reported that the survival rate without BPD at 36 weeks was higher in preterm infants receiving early hydrocortisone than in those not administered it (60% vs 51%; OR 1.48, 95% CI 1.02–2.16, p = 0.04) [90], although treatment had no effect on neurodevelopment [91]. Positive results were also obtained with low-dose dexamethasone (< 0.2 mg/kg/day), which is currently recommended for babies who remain ventilator dependent after 1–2 weeks [92]. A trial to evaluate whether even lower doses of dexamethasone (13 doses of 0.015 mL/kg/day in the first 16 days of life) is ongoing [93]. To reduce the risk of systemic GCS, inhaled GCS has been suggested and used in several trials. A recent meta-analysis of published trials concluded that inhaled steroids (beclomethasone, budesonide, fluticasone, flunisolide, and dexamethasone) were associated with a significant reduction in BPD development at 36 weeks of postmenstrual age (RR = 0.77, 95% CI 0.65–0.91) [94]. This finding seems to suggest that inhaled steroids are an obvious logical alternative to systemic steroids to prevent BPD. However, in the study by Bassler et al. a trend towards increased mortality in children receiving budesonide was shown [95]. Moreover, long-term effects on neurological development were not clearly established, indicating that, as for systemic steroids, further studies are needed to establish the doses and time of administration for inhaled steroids, together with the real risk of severe adverse events.

#### Caffeine

Caffeine is a methylxanthine derivative commonly used for the therapy of apnoea in preterm infants. The Caffeine for Apnea of Prematurity study showed that caffeine administration was associated with a significant reduction in BPD development and reduced neurodisability at 18 months of age [96, 97]. Recently, a number of cohort studies have further supported a role for caffeine in BPD prevention. Administration within the first 2 days of life seems to lead to a reduced risk of BPD compared to later administration (OR 0.69; 95% CI 0.58–0.82; p < 0.001), although it was found to be associated with an increased risk of necrotizing enterocolitis (OR 1.41; 95% CI 1.04–1.91; p = 0.027) [98–100]. The standard dose of caffeine citrate is 20 mg/kg for loading and 5–10 mg/kg for daily maintenance. The mechanism of action is unknown. However, it has been suggested that, considering its anti-inflammatory properties [101], it could play a role in reducing the pathogenic mechanisms of BPD development. However, it cannot be excluded that the positive effects might derive from the reduced need for assisted ventilation.

#### Vitamin A

Vitamin A is essential for normal development and the integrity of the respiratory tract. Because preterm infants have low vitamin A concentrations at birth, it was theorized that systematic supplementation of vitamin A might reduce the risk of BPD development. A recent meta-analysis of the studies published through May 2016 reported that, compared to children receiving placebo, children with EPT and VPT treated with vitamin A had a lower risk of BPD, defined as the need for oxygen supplementation at 36 weeks of gestational age [102]. However, although statistically significant, a reduction was observed only in children weighing < 1000 g and was small. Moreover, vitamin A does not reduce the mortality and duration of mechanical ventilation or length of hospital stay, and it does not improve neurodevelopmental outcomes at 18–22 months of age. Finally, the best route of supplementation is not definitively established, although the one trial that gave enteral vitamin A found no significant benefit for supplementation [103]. Consequently, it was suggested that the administration of vitamin A to preterm infants be balanced, considering advantages and problems. Administration is painful, must be repeated several times (i.e., 5000 U i.m. 3 times/week for a total of 12 doses) and has been associated with an increased risk of sepsis [104].

#### Nitric oxide

Inhaled nitric oxide has been found to be effective in the treatment of term neonates with acute hypoxaemic respiratory failure and persistent pulmonary hypertension [105]. However, the use of inhaled nitric oxide to prevent BPD development in preterm infants was not successful. A meta-analysis of the studies of this topic published through January 2016 found no advantages, either when the gas was administered early after birth or when it was administered later [106], which explains why routine use of nitric oxide is not recommended by experts and scientific societies [107, 108]. However, addition of vitamin A to nitric oxide administration can significantly improve final effect of nitric oxide inhalation [109]. Gadhia et al. [109] reported that the association of inhaled nitric oxide and intramuscular vitamin A reduced the incidence of BPD and BPD plus death in preterm infants weighing 750–999 g and improved neurocognitive outcomes at 1 years in those in the 500–749 birth weight group.

#### Cell therapy

There is evidence that stem and progenitor cell impairment can favour BPD development [110]. Based on this finding, it has been supposed that the use of exogenous stem or progenitor cells could protect or regenerate even a damaged lung.

A great number of animal studies have used lung-resident mesenchymal stem or progenitor cells (MSCs) in BPD models [111, 112]. Although MSCs precise role in organ function remains incompletely defined, mounting evidence suggested that they are an important component of the parenchymal progenitor cell niche and orchestrate organ homeostasis and repair following injury [111]. Different cell types—either intact cells or their conditioned medium—were administered, but bone marrow and umbilical cord blood derived mesenchymal stem cells were most prevalent. Available studies reported positive effects on outcome parameters including alveolar and vascular morphometry, lung function, and inflammation [112]. Cell homing to the lungs was demonstrated in some studies, but the therapeutic effects seemed to be mostly mediated via paracrine modulation of inflammation, fibrosis and angiogenesis. Positive results with increased vessel density, reduced pulmonary artery pressure and increased alveolar wall thickness were also obtained when MSCs derived from the bone marrow of healthy adult rodents were administered intravenously [113] or intratracheally [12]. Starting with these and other similar experimental results [114], attempts to evaluate cell therapy in humans have been undertaken. MSCs were found to be safe and well tolerated in adult patients [115–118]. No evidence of pro-fibrotic and tumorigenic potential of MSCs was reported. Moreover, in one study carried out in patients with chronic obstructive pulmonary disease and an elevated C reactive protein at baseline, it was demonstrated that MSC administration was followed by a significant reduction of the inflammatory marker [117].

The results of MSC administration in preterm infants were promising. Chang et al. assessed the safety and feasibility of a single, intratracheal transplant of human umbilical cord blood-derived MSCs in preterm infants with a mean gestational age of 25.3 ± 0.9 weeks old with high risk BPD [119]. Administration of both low and high doses of cells was well tolerated, whereas the concentration of pro-inflammatory cytokines in tracheal aspirates was significantly reduced after 7 days. Respiratory severity score measured in the 3 weeks after transplantation was significantly lower than in comparable historical controls [119]. Other studies are ongoing or have been completed (NCT02381366, NCT02443961, NCT01828957). Results have not yet been published. However, preliminary reports of the first of them [120] suggest that MSCs were generally well tolerated with no early deaths or cardiorespiratory problems. However, a study patient died about 4 months later as a result of pulmonary hypertension and lung hypoplasia [121]. All these findings clearly indicate that cell therapy could be an attractive measure for the future. However, further studies are needed to evaluate the role of MSCs in the pathogenesis and treatment of BPD. Which MCs have to be selected, the dose and duration of administration are not defined as the risk of adverse events following administration. Finally, large-scale production of MSCs for transplant is for the moment a very difficult problem to solve.

## Conclusions

BPD is a respiratory condition that presently occurs in EPT and VPT and can lead to chronic respiratory problems. Although knowledge about BPD pathogenesis has significantly increased in recent years, not all of the mechanisms that lead to lung damage are completely understood, which explains why therapeutic approaches that are theoretically effective have been only partly satisfactory or useless and, in some cases, potentially negative. However, prevention of prematurity, systematic use of nonaggressive ventilator measures, avoiding supraphysiologic oxygen exposure and administration of surfactant, caffeine and vitamin A can significantly reduce the risk of BPD development. Cell therapy is the most fascinating new measure to address the lung damage due to BPD. It is desirable that ongoing studies yield positive results to definitively solve a major clinical, social and economic problem.

## Abbreviations

BPD: bronchopulmonary dysplasia
CI: confidence interval
CT: computed tomography
DALY: disability-adjusted life year
ELBW: extremely low birth weight infants
EPT: extremely preterm
FVC: forced vital capacity
FEV1: forced expiratory volume in 1 s
FEF25%–75%: forced expiratory flow between 25 and 75% of vital capacity
GCS: glucocorticoids
HFNCs: high flow nasal cannulas
HRCT: high-resolution computed tomography
IL: interleukin
MPT: modestly preterm infants
MSCs: mesenchymal stem or progenitor cells
OR: odds ratio
NCPAP: nasal continuous positive airway pressure
NIPPV: non-invasive positive pressure ventilation
PaCO_2_: partial pressure of CO_2_ in the arterial blood
ROS: reactive oxygen species
RR: relative risk
RSV: respiratory syncytial virus
SD: standard deviation
Th2: T-helper 2
VPT: very preterm infants
